# *α*-Fetoprotein and human chorionic gonadotrophin-*β* as prognostic markers in neuroendocrine tumour patients

**DOI:** 10.1038/sj.bjc.6604428

**Published:** 2008-06-24

**Authors:** T Shah, R Srirajaskanthan, M Bhogal, C Toubanakis, T Meyer, A Noonan, C Witney-Smith, T Amin, P Bhogal, N Sivathasan, B Warner, D Hochhauser, M E Caplin

**Affiliations:** 1 Neuroendocrine Tumour Unit, Centre for Gastroenterology, Royal Free Hospital, Pond Street, London, NW3 2QG, UK; 2 UCL Cancer Institute, University College London, Gower Street, London, WC1E 6BT, UK

**Keywords:** neuroendocrine tumours, tumour marker, *α*-fetoprotein, AFP, human chorionic gonadotrophin subunit *β*, hCG*β*, ChA, Ki-67/MIB-1

## Abstract

Serum chromogranin A is the most useful general and prognostic tumour marker available for neuroendocrine tumour (NET) patients. The role of other tumour markers is less clear. In order to determine the diagnostic and prognostic value of serum *α*-fetoprotein (AFP) and human chorionic gonadotrophin-*β* (hCG*β*) in NETs, a database containing biochemical, histological, and survival data on 360 NET patients was constructed. This data was statistically assessed, using Statistical Package for the Social Sciences, to determine the utility of commonly measured tumour markers with particular emphasis on AFP and hCG*β*. *α*-Fetoprotein and hCG*β* were raised in 9.5 and 12.3% of patients respectively and jointly raised in 9.1% of patients in whom it was measured. *α*-Fetoprotein levels associated strongly and positively with tumour grade, serum CgA and hCG*β* levels, and worse survival. Human chorionic gonadotrophin-*β* levels also associated strongly and positively with serum CgA and AFP levels, and worsening survival. *α*-Fetoprotein and hCG*β* are elevated in high-grade NETs, with a rapidly progressive course and poorer survival. They also correlate with chromogranin-A, which is known to be a marker of tumour burden and to have prognostic value. Thus AFP and hCG*β* are clinically important in NETs and when elevated are poor prognostic markers.

Neuroendocrine tumours (NETs) comprise a heterogeneous group of tumours classified as being either functional, with symptoms due to hormone secretion, or nonfunctional due to an apparent lack of hormone-associated symptoms (Solcia *et al*, 2000). The functional tumours may produce specific hormones, the measurement of which can aid diagnosis and management; for example, serotonin with midgut carcinoid tumours, insulin with insulinoma, and gastrin in gastrinoma. Currently, the most useful ‘general’ NET marker is plasma CgA, and recent guidelines are recommending that all patients with NETs should have their CgA measured (Ramage *et al*, 2005; Rindi *et al*, 2006a).

Tumour markers can provide valuable information on tumour functionality and presumed tumour burden in relation to antitumour therapy. For example, an indolent nonfunctioning NET may produce increased amounts of CgA as the first sign of a switch to a more progressive course. Alternatively, antitumour therapy may not display tumour response as determined radiologically, using the RECIST criteria, as tumour cells may be replaced by fibrosis with a resultant lack of change in overall tumour size. However, there may be a significant decline in the level of circulating tumour markers together with significant symptomatic improvement. In addition, tumour markers can have prognostic value: plasma neurokinin-A has been shown to be an accurate marker of prognosis in midgut NETs, with rising neurokinin-A levels despite somatostatin analogue therapy shown to be associated with poorer prognosis (Turner *et al*, 2006); patients with CgA greater than 5000 *μ*g l^−1^ have a 5-year survival of only 22% as opposed to 63% for patients with serum chromogranin-A levels of less than 5000 *μ*g l^−1^ (Janson *et al*, 1997). Absolute plasma CgA levels may also help to differentiate between localised and diffuse NET spread, as well as between chronic active gastritis and NETs (Campana *et al*, 2007).

There is a continuing need for tumour markers that can provide further diagnostic and prognostic information in NET patients. *α*-Fetoprotein (AFP) and human chorionic gonadotrophin-*β* (hCG*β)* have previously been reported as being elevated in some NET patients; however, the utility of these markers in NET patients has not been determined (Ramage *et al*, 2005). Lokich *et al* (1987) reported, in a small series, that AFP may be raised in a small proportion of NET patients and that it may provide some useful information. However, this initial report has not been further explored. *α*-Fetoprotein is reactivated in numerous cancers, including hepatocellular carcinoma and teratocarcinomas. The basis of this reactivation is not well understood, but may involve changes in the level or activity of transcriptional regulators (Spear, 1999).

Human chorionic gonadotrophin-*β* belongs to the glycoprotein hormone family that also comprises luteinising hormone (LH), follicle-stimulating hormone, and thyroid-stimulating hormone. All members are heterodimers consisting of an *α-* and a *β*-subunit. The *α*-subunit, which is common to all glycoprotein hormones, contains 92 amino acids. The *β*-chains determine the biological activity and display extensive homology, with that between hCG*β* and LH*β* being about 80%. Human chorionic gonadotropin is mainly used for detection and monitoring of pregnancy and pregnancy-related disorders, and as an extremely sensitive and specific marker for trophoblastic tumours of placental and germ cell origin (Stenman *et al*, 2006).

The aims of this study were to determine the role of AFP and hCG*β* as diagnostic and prognostic markers, their relationship with other tumour markers, as well as their value in predicting disease progression in NET patients.

## Patients and methods

We have developed a database of NET patients, containing biochemical, radiological, histological, and survival data available for 360 patients. Patients had been consented for approval of utilisation of blood and tissue results for research purposes. Pretreatment biochemical data included tumour markers AFP, hCG*β*, and CgA. Histology data were collected to determine tumour grade. Neuroendocrine tumour histological grading was assessed according to the new TNM classification, including differentiation of tumour and proliferation index Ki-67 (MIB-1): thus classified as low-grade (G1) mitotic count <2/10 high-powered fields (HPF) or Ki-67 ⩽2%; intermediate-grade (G2) mitotic count 2–20/10 HPF or Ki-67 3–20%; and high-grade (G3) mitotic count >20/10 HPF or Ki-67 >20% (Rindi *et al*, 2006b). Of the 360 patients in the database, 294 had been tested for AFP. Of these, 294 patients, a subset of 28 patients, was identified with serum AFP levels at least 1.5 × the upper limit of normal. A further 40 patients with normal AFP were randomly chosen from the database to act as an age- and sex-matched control for the AFP-high patients. In all, 268 patients had been tested for hCG*β*, of which 33 patients had hCG*β* levels at least 1.5 × the upper limit of normal. A further 33 patients with normal hCG*β* were randomly chosen from the database to act as an age- and sex-matched control for the hCG*β*-high patients.

A subset of 21 patients was derived from the AFP and hCG*β* groups that had raised AFP and hCG*β* in combination. This subset comprised 9.1% of patients in whom the two markers had been measured. Control group had normal serum AFP and hCG*β* levels.

**Figure 1:**
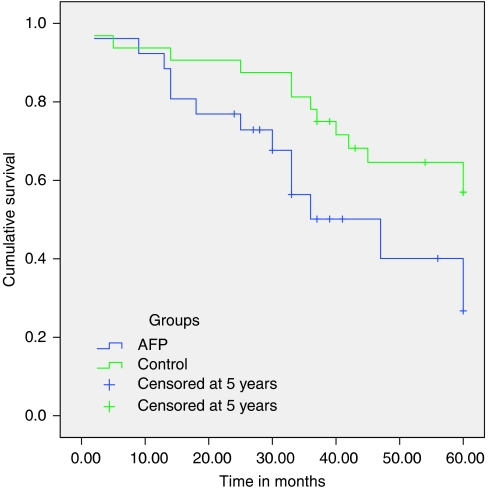
Kaplan–Meier graph comparison of 5-year survival between the high-AFP and control (normal-AFP) groups (*P*=0.001).

**Figure 2:**
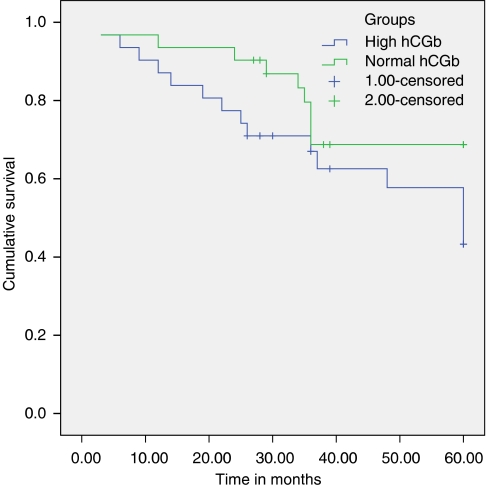
Kaplan–Meier graph comparison of 5-year survival between the high-hCGb and control (normal-hCGb) groups (*P*=0.037).

**Figure 3:**
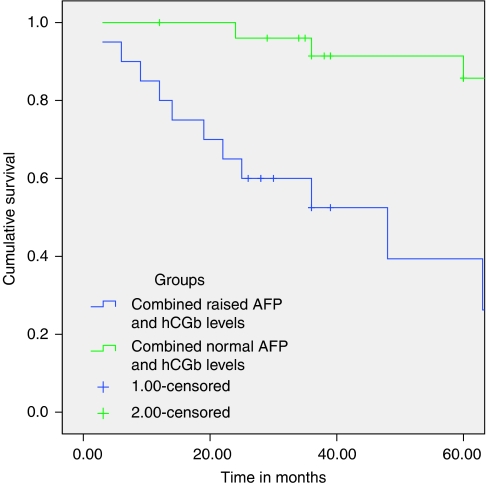
Kaplan–Meier graph comparison of 5-year survival between the combined high-AFP/hCGb and control (normal-AFP/hCGb) groups (*P*=0.001).

Statistical Package for the Social Sciences was utilised to determine any difference between the two tumour marker groups and their respective controls (Mann–Whitney *U*-test for nonparametric samples; Tables 1, 3 and 5); to discover any correlations between the parameters recorded (Spearman's *ρ*; Tables 2, 4 and 6); and to look for survival differences (Kaplan–Meier survival curves; Figures 1, 2, 3).

## Results

### AFP group

Twenty-eight out of 294 patients (9.5%) had elevated AFP. The AFP-high and control groups were compared using Mann–Whitney *U*-test, for two nonparametric samples, to look for significant differences in age and gender makeup of the two groups. This confirmed the two groups to be evenly matched for age and sex (Table 1). There were significant differences between the two groups for the measured tumour markers AFP, hCG*β*, CgA, MIB-1 (Table 1), and survival as measured from the time of diagnosis (Figure 1; Table 1).

The data from the two groups were then combined for further statistical analysis to determine possible correlations between the five parameters studied: absolute levels of AFP, MIB-1, hCG*β*, CgA and patient survival. Spearman's *ρ* test was applied to the data (Table 2). Serum AFP levels were discovered to correlate with the four parameters measured. Thus, rising serum AFP levels strongly and positively correlated with rising hCG*β* and CgA levels, and MIB-1 scores. There was an additional strongly negative correlation with survival from the time of diagnosis (significant at the 0.01 level) (Table 2).

### Human chorionic gonadotrophin-*β* group

Thirty-three out of 268 patients (12.3%) had elevated hCG*β*. The hCG*β*-high and control groups were compared using Mann–Whitney *U*-test, for two nonparametric samples, to look for significant differences in age and gender makeup of the two groups. This confirmed the two groups to be evenly matched for age and sex (Table 3). There were significant differences between the two groups for the measured levels of tumour markers hCG*β*, AFP, CgA, and survival as measured from the time of diagnosis (Table 3; Figure 2), but not for MIB-1 scores (Table 3).

The data from the two groups were then combined for further statistical analysis to determine possible correlations between the five parameters studied: absolute levels of hCG*β*, AFP, MIB-1, CgA, and survival from time of diagnosis. Spearman's *ρ* test was applied to the data (Table 4).

Serum hCG*β* levels were discovered to be correlated with three of four other parameters measured. That is, rising serum hCG*β* levels strongly and positively correlated with rising AFP and CgA levels. Rising hCG*β* levels negatively correlated with survival from the time of diagnosis (significant at the 0.01 level) (Table 4). No correlation was found between hCG*β* levels and MIB-1 score.

### Combined AFP/hCG*β* group

Twenty-one out of 230 patients (9.1%) had combined elevation of serum AFP and hCG*β*. The combined AFP/hCG*β*-high and control groups were compared using Mann–Whitney *U*-test, for two nonparametric samples, to look for significant differences in age and gender makeup of the two groups. This confirmed the two groups to be evenly matched for age and sex (Table 5). There were significant differences between the two groups for the measured levels of tumour markers hCG*β*, AFP, CgA, and survival as measured from the time of diagnosis (Table 5; Figure 3), but not for MIB-1 scores (Table 5).

The data from the two groups were then combined for further statistical analysis to determine possible correlations between the five parameters studied: absolute levels of hCG*β*, AFP, MIB-1, CgA, and survival from time of diagnosis. Spearman's *ρ* test was applied to the data (Table 6).

These statistical tests discovered the serum AFP levels to correlate with the four parameters measured. That is, rising serum AFP levels strongly and positively correlated with rising hCG*β* and CgA levels, and MIB-1 scores; rising serum AFP strongly and negatively correlated with survival from the time of diagnosis (significant at the 0.01 level) (Table 6). Serum hCG*β* levels correlated with three of four other parameters measured. That is, rising serum hCG*β* levels strongly and positively correlated with rising AFP and CgA levels. Rising hCG*β* levels negatively correlated with survival from the time of diagnosis (significant at the 0.01 level) (Table 6). No correlation was found between hCG*β* levels and MIB-1 score (Table 6).

## Discussion

A number of putative tumour markers are measured in NET patients, with CgA having the highest expression in NETs and being considered the most useful diagnostic and prognostic marker (Janson *et al*, 1997; Turner *et al*, 2006; Campana *et al*, 2007). Tumour markers are measured at regular intervals and may be useful for monitoring disease progression. Often a rise in tumour marker levels may precede clinical indicators of disease progression such as worsening diarrhoea, facial flushing, and weight loss, as well as objective indicators of disease progression as determined radiologically. Furthermore, an increase in the number of expressed tumour markers is associated with worsening prognosis (Ardill and Erikkson, 2003).

To date, there is only limited data on most tumour markers measured in NET patients, which has thus created uncertainty about their role. Clinical impression of a linkage between high-grade aggressive tumours and a rise in serum AFP and hCG*β* levels led us to perform a systematic review of the utility of AFP and hCG*β* measurement in our NET patients. Although, very little is known about AFP in NETs, some useful data already exist on the expression and value of *α*- and *β-*subunits of hCG (Heitz *et al*, 1987; Eriksson *et al*, 1989; Grossmann *et al*, 1994; Nobels *et al*, 1997). These subunits have been shown to be raised in a significant proportion of NET patients and to have the ability to differentiate between benign and malignant gastroenteropancreatic tumours (Heitz *et al*, 1987; Eriksson *et al*, 1989; Grossmann *et al*, 1994). However, neither AFP nor subunits of hCG are able to differentiate between NETs and other tumours (Nobels *et al*, 1997; Yuen and Lai, 2005).

This analysis of a large NET patient database demonstrates, for the first time, the ability of two easily measurable agents to prognosticate in NET patients. Both AFP and hCG*β* are shown to be related to poorer survival, with the clearest difference being seen between the group of patients with combined rise in AFP and hCG*β* in comparison to controls matched for age at diagnosis and sex. However, only AFP associated with Ki-67. This can be explained by the fact that there are significant differences in the disease stage between the AFP-high group and its control group, whereas no significant differences in the disease stage existed between the hCG*β*-high group and its control group (Tables 1 and 3).

Although overall AFP is elevated only in a minority of NET patient, this data analysis demonstrates the ability of AFP to highlight a group of NET patients with aggressive, high-grade tumours and poor prognosis. Interestingly, four patients with raised AFP did not have liver metastases but did have large volume disease elsewhere (neck, peritoneum, and chest). Thus AFP is likely to be a marker of tumour cell de-differentiation rather than a marker of hepatic metastases from NETs.

Human chorionic gonadotrophin-*β* also provides prognostic information with its demonstrated correlation with impaired survival and other markers of a poor outcome, namely CgA and AFP. When hCG*β* is high, as defined by levels >1.5 × upper limit of normal, the Mann–Whitney *U*-test applied to these values demonstrates a clear difference in survival between the two groups (Table 3). On determining a correlation between the absolute hCG*β* level and degree of impairment of survival, although there is a trend to impaired survival this is not statistically significant (Table 4). The results of these analyses demonstrate that an abnormally high hCG*β* is more informative than the absolute level itself. Thus hCG*β*, when raised, is a marker of poor prognosis (Table 3, Figure 2).

The clearest predictions, however, can be made for those patients with a combined rise in both serum AFP and hCG*β* levels. These are strongly associated with high CgA levels and worsening prognosis. Conversely, patients with normal AFP and hCG*β* levels have low serum CgA levels and an excellent 5-year prognosis (Figure 3).

Most NET patients have extensive metastases at the time of diagnosis. The indolent nature of these tumours means that even in the presence of liver metastases, the 5-year survival rates are surprisingly good. However, a proportion of patients have rapidly progressive disease, at diagnosis, which requires aggressive management with cytoreductive therapies, hopefully resulting in better symptom control and improved survival. Predicting the behaviour of NETs in individual cases has to date relied on tumour histology, serum CgA levels, and serial radiology imaging. This data clearly demonstrate the utility of AFP, hCG*β*, CgA, and possibly Ki-67 index in highlighting those patients with WHO stage IV disease who are going to require intensive monitoring and possibly early and aggressive therapy. Conversely, patients with favourable results can be re-assured about their medium- to long-term survival and monitored less intensely with confidence, perhaps six monthly as opposed to three to four monthly for those patients with, for example, elevated AFP and hCG*β*.

This is a retrospective study and the results highlight the worth of performing a prospective study to assess these markers.

In conclusion, this study has identified AFP and hCG*β* to be capable of providing significant prognostic information relevant to the management of NET patients.

## Figures and Tables

**Table 1:** Comparison of demographic and tumour marker data for the raised-AFP and control groups demonstrating the two groups to be evenly matched for age, gender, and diagnosis

	**High AFP**	**Control**	***P*-value (Mann–Whitney *U*-test)**
Age at diagnosis	51	48	0.597
Sex (M/F)	15/13	18/22	0.495
			
*Diagnosis*			
Metastatic NET (of unknown primary)	9	13	
Pancreatic NET	11	16	
Gastrinoma	1	2	
Bronchial	2	4	
Midgut NET	1	3	
Hindgut NET	1	0	
MTC	1	1	
Thymic NET	1	1	
Pelvic NET	1	0	
Mean AFP (0–11.3 ng ml^−1^)	273.8	3.5	0.001
Mean hCG*β* (<2.5 mIU ml^−1^)	21.3	1.6	0.001
Mean CgA (0–60 U l^−1^)	423	113	0.002
Mean Ki-67 (MIB-1)	21	10	0.009
Mean survival (months)	37.6	69	0.001
Stage IV disease (WHO classification)	24 of 28 (86%)	25 of 40 (63%)	0.037

AFP=*α*-fetoprotein; hCG*β*=human chorionic gonadotrophin-*β* ; NET=neuroendocrine tumour.

Significant differences between the two groups are apparent for the expression of tumour markers AFP, hCG*β*, CgA, Ki-67 score, and survival from the time of diagnosis.

**Table 2:** Correlation of AFP levels with Ki-67 score, serum hCG*β*, and CgA levels, as well as survival in NET patient

			**Ki-67 (MIB-1)**	**hCG*β***	**CgA**	**Survival in months**
Spearman's *ρ*	AFP	Correlation coefficient	0.381 (^**^)	0.558 (^**^)	0.451 (^**^)	−0.419 (^**^)
		Significant (two-tailed)	0.007	0.000	0.000	0.001

AFP=*α*-fetoprotein; hCG*β*=human chorionic gonadotrophin-*β* ; NET=neuroendocrine tumour.

^**^Correlation is significant at the 0.01 level (two-tailed).

**Table 3:** Comparison of demographic and tumour marker data for the raised-hCG*β* and control groups demonstrating the two groups to be evenly matched for age, gender, and diagnosis

	**High hCG*β***	**Control**	***P*-value (Mann–Whitney *U* -test)**
Age at diagnosis	55	54	0.891
Sex (M/F)	16/17	16/17	
			
*Diagnosis*			
Metastatic NET (of unknown primary)	8	7	
Pancreatic NET	15	11	
Gastrinoma	2	1	
Bronchial	4	5	
Midgut NET	1	6	
Hindgut NET	1	1	
MTC	1	1	
Thymic NET	1	1	
Mean hCG*β* (<2.5 mIU ml^−1^)	198.7	2.0	0.001
Mean AFP (0–11.3 ng ml^−1^)	238.1	7.9	0.001
Mean CgA (0–60 U l^−1^)	414.2	180.3	0.02
Mean Ki-67 (MIB-1)	13	14	0.63
Mean survival (months)	48	57.3	0.037
Stage IV disease (WHO classification)	27 of 33 (82%)	24 of 33 (73%)	0.382

AFP=*α*-fetoprotein; hCG*β*=human chorionic gonadotrophin-*β* ; NET=neuroendocrine tumour.

Significant differences between the two groups are apparent for the expression of tumour markers AFP, hCG*β*, CgA, and survival from the time of diagnosis.

**Table 4:** Correlation of hCG*β* levels with AFP and CgA levels

			**Ki-67 (MIB-1)**	**AFP**	**CgA**	**Survival in months**
Spearman's *ρ*	hCG*β*	Correlation coefficient	0.012	0.634 (^**^)	0.291 (^*^)	−0.229
		Significant (two-tailed)	0.931	0.000	0.019	0.074

AFP=*α*-fetoprotein; hCG*β*=human chorionic gonadotrophin-*β* ; NET=neuroendocrine tumour.

There is also a trend towards a statistically significant correlation between hCG*β* levels and survival, but clear absence of linkage between hCG*β* levels and Ki-67 score.

^**^Correlation is significant at the 0.01 level (two-tailed).

^*^Correlation is significant at the 0.05 level (two-tailed).

**Table 5:** Comparison of demographic and tumour marker data for the combined raised serum hCG*β*/AFP levels and control groups demonstrating the two groups to be evenly matched for age, gender, and diagnosis

	**Combined raised AFP and hCG*β***	**Control**	***P* value (Mann–Whitney *U* -test)**
Age at diagnosis	55.4	55.4	0.691
Sex (M/F)	13/8	15/13	
			
*Diagnosis*			
Metastatic NET (of unknown primary)	6	6	
Pancreatic NET	10	10	
Gastrinoma	0	0	
Bronchial	2	5	
Midgut NET	0	5	
Hindgut NET	1	1	
MTC	1	2	
Thymic NET	1	1	
Mean AFP (0–11.3 ng ml^−1^)	371.7	35.4	0.001
Mean hCG*β* (<2.5 mIU ml^−1^)	283.6	2.2	0.001
Mean CgA (0–60 U l^−1^)	511	210	0.014
Mean Ki-67 (MIB-1)	15.6	11.9	0.283
Mean survival (months)	29.9	61.2	0.001
Stage IV disease (WHO classification)	18 of 21 (86%)	20 of 28 (71%)	0.240

AFP=*α*-fetoprotein; hCG*β*=human chorionic gonadotrophin-*β* ; NET=neuroendocrine tumour.

Significant differences between the two groups are apparent for the expression of tumour markers AFP, hCG*β*, CgA, and survival from the time of diagnosis.

**Table 6:** Correlation of AFP levels with Ki-67 score, serum hCG*β*, and CgA levels, as well as survival in NET patient

			**Ki-67 (MIB-1)**	**AFP**	**hCGb**	**CgA**	**Survival in months**
Spearman's *ρ*	AFP	Correlation coefficient	0.330 (^*^)	1.000	0.778 (^**^)	0.413 (^**^)	−0.486 (^**^)
		Significant (two-tailed)	0.035		0.000	0.004	0.001
Spearman's *ρ*	hCGb	Correlation coefficient	0.090	0.778 (^**^)	1.000	0.310 (^*^)	−0.410 (^**^)
		Significant (two-tailed)	0.575	0.000		0.032	0.005

AFP=*α*-fetoprotein; hCG*β*=human chorionic gonadotrophin-*β*; NET=neuroendocrine tumour.

^**^Correlation is significant at the 0.01 level (two-tailed).

^*^Correlation is significant at the 0.05 level (two-tailed).
